# Iteratively forecasting biological invasions with PoPS and a little help from our friends

**DOI:** 10.1002/fee.2357

**Published:** 2021-06-03

**Authors:** Chris M Jones, Shannon Jones, Anna Petrasova, Vaclav Petras, Devon Gaydos, Megan M Skrip, Yu Takeuchi, Kevin Bigsby, Ross K Meentemeyer

**Affiliations:** ^1^ Center for Geospatial Analytics North Carolina State University Raleigh NC; ^2^ Animal and Plant Health Inspection Service (APHIS) US Department of Agriculture (USDA) Riverdale MD; ^3^ Center for Integrated Pest Management North Carolina State University Raleigh NC; ^4^ APHIS USDA Raleigh NC; ^5^ Department of Forestry and Environmental Resources North Carolina State University Raleigh NC

## Abstract

Ecological forecasting has vast potential to support environmental decision making with repeated, testable predictions across management‐relevant timescales and locations. Yet resource managers rarely use co‐designed forecasting systems or embed them in decision making. Although prediction of planned management outcomes is particularly important for biological invasions to optimize when and where resources should be allocated, spatial–temporal models of spread typically have not been openly shared, iteratively updated, or interactive to facilitate exploration of management actions. We describe a species‐agnostic, open‐source framework – called the Pest or Pathogen Spread (PoPS) Forecasting Platform – for co‐designing near‐term iterative forecasts of biological invasions. Two case studies are presented to demonstrate that iterative calibration yields higher forecast skill than using only the earliest‐available data to predict future spread. The PoPS framework is a primary example of an ecological forecasting system that has been both scientifically improved and optimized for real‐world decision making through sustained participation and use by management stakeholders.

**In a nutshell:**

- Because *where* something happens is just as important to anticipate as *when*, ecological forecasts should predict changes across both space and time
- Resource managers must be co‐creators of spatial ecological forecasting systems to optimize their use for real‐world management decisions
- We developed a forecasting system in collaboration with partners at the US Department of Agriculture to predict the spread of pests and pathogens, pairing a user‐friendly interface with a flexible spread model that can be used for any species
- Our system exemplifies a strategy to make near‐term ecological forecasts actionable for environmental decision making

Ecological forecasting represents a paradigm shift in ecology focused on making repeated, testable predictions across management‐relevant timescales, geographies, and contexts to test scientific assumptions and predict near‐future conditions (Clark *et al*. 2001; Dietze *et al*. 2018; Dietze and Lynch 2019). Over the past decade, model forecasts have been developed to predict species distributions, fisheries harvests, insect phenology (Crimmins *et al*. 2020), pests and pathogen risk (McMullen *et al*. 2012), and more (eg see references in Dietze *et al*. 2018), and best practices have been codified for the development and use of such forecasts (Harris *et al*. 2018; Hobday *et al*. 2019). However, ecological forecasting has yet to reach its full potential for serving resource management because (1) researchers and decision makers rarely co‐produce forecasts, (2) forecasters do not incorporate interactive decision analytics to explore how management actions may affect future conditions, and (3) spatially explicit modeling approaches for predicting patterns of change across geographic areas have not been adopted.

Involving decision makers and other stakeholders in the co‐development of models (Voinov and Bousquet 2010) and the design of forecasting systems (Hobday *et al*. 2016; Gaydos *et al*. 2019) increases transparency, trust, and relevance. Advocates of ecological forecasting emphasize the importance of co‐production between modelers and domain experts (eg Payne *et al*. 2017; Dietze *et al*. 2018), but the inherent challenges of knowledge co‐production often preclude the formation of productive partnerships between resource managers and research scientists to co‐develop a research agenda (Djenontin and Meadow 2018). Accordingly, many forecasts produce a quantitative output that can alert resource managers about future conditions (eg GCC 2020), but few allow stakeholders to interactively explore the effects that management actions may have on a forecast.

Scenario exploration can be facilitated by computational tools known as decision analytics: interactive, user‐friendly computer interfaces that allow resource management experts, and not necessarily modeling experts, to easily modify model simulations by specifying realistic management actions (Voinov *et al*. 2016; Tonini *et al*. 2017; Gaydos *et al*. 2019). Some forecasting frameworks report predictions online (eg usanpn.org/data/forecasts; Crimmins *et al*. 2020) or permit interaction through web‐based systems to tailor reporting of results (eg www.wheatscab.psu.edu; Cunniffe *et al*. 2015b; NOAA 2019), but to the best of our knowledge none explicitly permit on‐the‐fly testing of where and when to apply interventions that resource managers may want to simulate, thereby predicting impacts before expending resources or making (in hindsight) deleterious choices in the real world.

Resource management requires complex decision making across space and time, necessitating forecasts that are spatially explicit (ie those that take into account the locations of events or entities and the geographic relationships between them). Spatially explicit forecasting is particularly important for biological invasions, because *where* a new infestation or infection will occur is just as important to anticipate as *when* it will occur. Research teams have been modeling the spread of pests and pathogens both spatially and temporally for many years (eg Kampmeijer and Zadoks 1977; Fitzpatrick *et al*. 2012; Cunniffe *et al*. 2016), but these efforts have not heeded the call of Dietze *et al*. (2018) to iteratively update model parameters with new data or openly share forecasts to build a community of forecasting practice. Nor do they integrate decision analytics that support stakeholder interaction with the models, as was suggested by Cunniffe *et al*. (2015a,b).

To meet these challenges, we developed a species‐agnostic forecasting framework called the Pest or Pathogen Spread (PoPS) Forecasting Platform, which to the best of our knowledge is the first example of a flexible, open‐source platform for near‐term (≤ 5 years) iterative ecological forecasting that combines several key features typically missing in ecological models. For example, (1) PoPS was designed, and continues to be developed, through a collaborative, participatory process with government analysts and field operations personnel; (2) the system features interactive decision analytics for generating and testing management interventions; and (3) the underlying spread model is both dynamic (accounts for changes over time) and spatially explicit (incorporates relationships and changes across space). We developed PoPS specifically for use by management professionals who do not necessarily have modeling experience and tailored it for predicting the spread, containment, and control of biological invasions.

Here, we describe the PoPS Forecasting Platform and its use for controlling biological invasions. Our approach emphasizes the value of co‐designing an ecological forecasting system that is modifiable for widespread use by management professionals without modeling expertise. We illustrate the potential of PoPS for improving management outcomes using two case studies, one involving a pest (spotted lanternfly [SLF], *Lycorma delicatula*) and the other a pathogen (the water mold that causes sudden oak death [SOD], *Phytophthora ramorum*), and show how iterative calibration with new data improves model skill as measured by accuracy assessments that capture spatial patterns.

## Ecological forecasting to manage biological invasions

Worldwide, biological invasions by pests and pathogens threaten food security (Paini *et al*. 2016; Savary *et al*. 2019), biodiversity (Simberloff *et al*. 2013), and ecosystem function (Lovett *et al*. 2006; Aukema *et al*. 2010), and represent a new frontier in ecological forecasting (Dietze *et al*. 2018). Spatial–temporal models are useful for understanding spread dynamics (eg Meentemeyer *et al*. 2011; Fitzpatrick *et al*. 2012) and exploring management interventions (eg Cunniffe *et al*. 2016), but they have typically been the domain of academic specialists and are rarely updated with new information to incorporate improved understanding of the system or validate their predictive capacity. Given the dynamic nature of spread, efforts to contain and control invasions require flexible, accessible tools to support decision makers; ecological forecasting presents the opportunity to test and explore management scenarios in a complex decision‐making space.

Practitioners facing an emerging invasion must make decisions concerning not only what should be done to contain the pest or pathogen but also when and where interventions would be most effective. One agency concerned with developing new tools to improve on‐the‐ground management of biological invasions is the US Department of Agriculture (USDA) Animal and Plant Health Inspection Service (APHIS), which partnered with our research group at North Carolina State University to co‐develop a spatially explicit spread forecasting system to support their operations.

**Figure 1:**
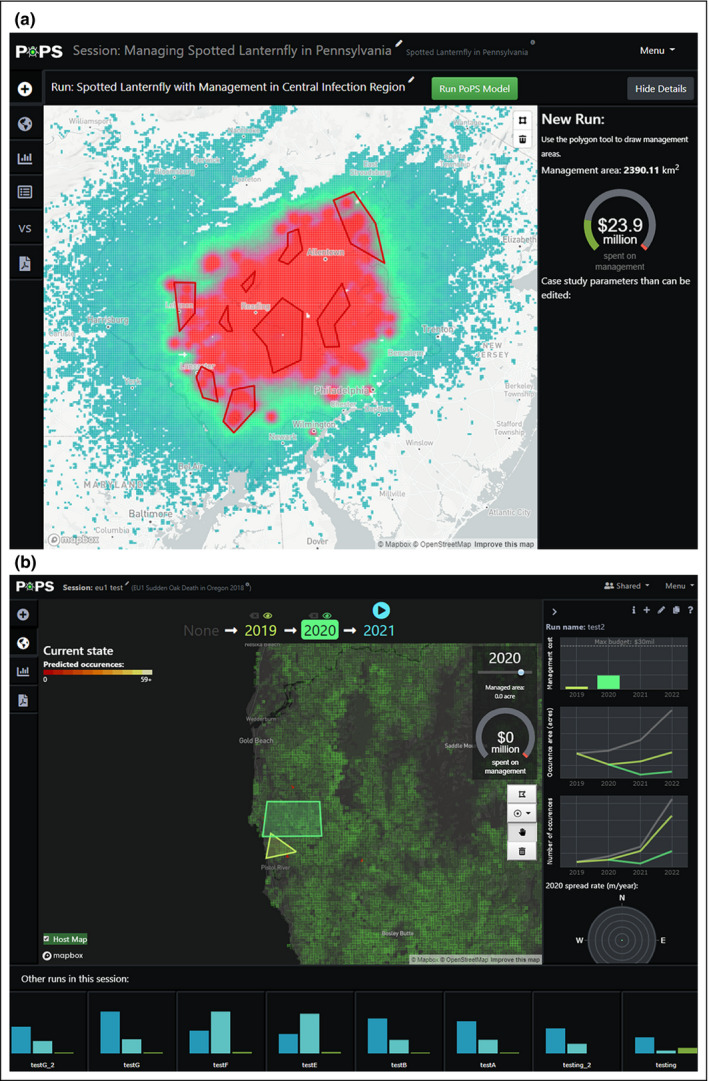
Stakeholder engagement improved the Pest or Pathogen Spread (PoPS) Forecasting Platform’s web‐based dashboard by providing input on interactive analytics most useful for management. Three features in (a) an early version of the dashboard were expanded in (b) the current version to include: (1) adaptive management in multiple years, (2) custom drawing tools allowing managers to easily add pre‐defined management areas, and (3) a comprehensive breakdown of budget. Three new features were added at stakeholders’ request: (4) a side panel with detailed analytics, (5) a panel for quickly comparing management strategies, and (6) a time slider showing field observations or forecasted data, depending on the selected year.

The system we developed, PoPS, is open‐source, scalable, and replicable by other research groups. All results presented below can be replicated through the PoPS web interface (https://popsmodel.org), and the decision analytics we designed can be built using the code available on GitHub (https://osf.io/q32p9) (Jones *et al*. 2020). Our collaboration with APHIS, which is maintained through biweekly meetings and semi‐annual workshops, has influenced PoPS’ web‐based interface design (Figure 1); led to the creation of user‐friendly decision analytics; and resulted in the development of a customizable species‐agnostic model that takes into account the factors that impact a pest or pathogen’s reproduction, dispersal, and establishment (Panel 1; Meentemeyer *et al*. 2011).

Panel 1 **The PoPS model**

**Figure 2:**
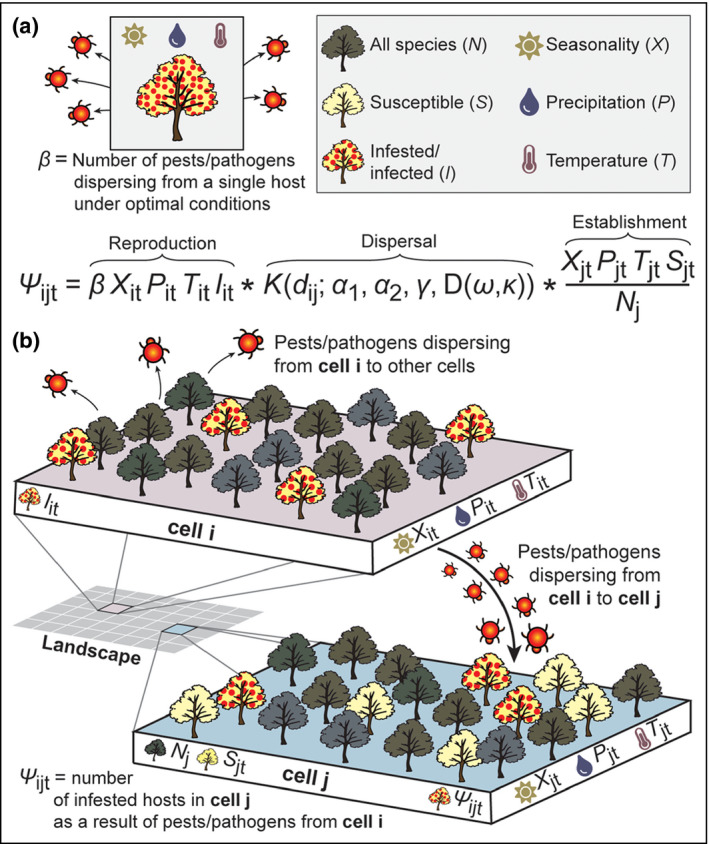
(a) Beta (β), the number of pests or pathogens that disperse from a single host under optimal environmental conditions, is the starting point of the PoPS model, which can be conceptualized in terms of reproduction, dispersal, and establishment. (b) Spread across a landscape is predicted by calculating infestation or infection (Ψ) for each cell.

The flexible, customizable Pest or Pathogen Spread (PoPS) Forecasting Platform simulates reproduction, dispersal, and establishment of pests and pathogens through space and time. For every location in a landscape, at each time step, the model predicts the number of infested or infected hosts (Ψ). The model is conceptually driven, first, by the notion of beta (β; Figure 2a). Because conditions are rarely optimal and locations contain multiple hosts, β is modified by the number of currently infested or infected hosts (*I*) and environmental conditions in a location (*i*) at a particular time (*t*) to determine reproduction. The dispersal kernel then determines where the new dispersing propagules go; dispersal distance (*d*) is a function of gamma (γ), which indicates how much dispersal is short‐distance (alpha‐1, α_1_) or long‐distance (alpha‐2, α_2_). The distance of each propagule is determined by drawing from a distribution using either α_1_ or α_2_, and its direction is drawn from a distribution that accounts for predominant wind direction (ω) and wind strength (κ). Once a propagule has landed in a new location, its establishment depends on the environmental conditions in that new location (*X*, *P*, *T*) and the availability of suitable hosts, calculated as the number of susceptible hosts (*S*) divided by the total number of potential hosts (*N*). Each application of PoPS uses a customized host map that provides the locations of target hosts in each grid cell across the modeled landscape. The value of Ψ (Figure 2b) is predicted for each cell, forecasting the spread of a pest or pathogen from infested or infected hosts to susceptible hosts, among all cells, across the landscape. The model runs quickly, even for landscapes with millions of cells.

PoPS is a modular, spatially explicit, discrete‐time model, meaning that various components (eg weather effects or long‐range dispersal) can be included or excluded from the model as necessary (via intuitive on–off switches on the interface) depending on the drivers that influence the species of interest. In addition, the model accounts for spatial relationships and movements between grid cells in a landscape, and forecasts across sequential time steps, which can be specified as daily, weekly, monthly, or yearly (Panel 1; Figure 2).

**Figure 3:**
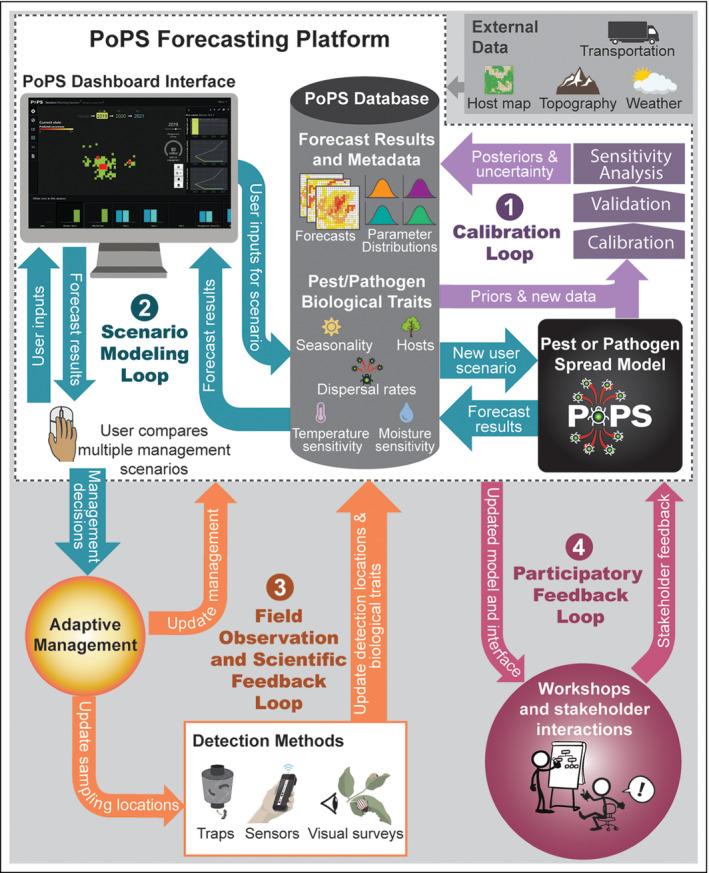
The iterative modeling cycle. (1) Calibration Loop, (2) Scenario Modeling Loop, (3) Field Observation and Scientific Feedback Loop, and (4) Participatory Feedback Loop. See text for details.

PoPS was developed, and continues to be refined, through an “iterative modeling cycle” (Figure 3), a process we suggest may help guide ecological forecasters in other contexts. Meeting with the stakeholders who will use the forecasts comes first, to discuss their needs and set mutual objectives. After initial data are gathered to feed into the model, four revisionary loops follow: (1) the Calibration Loop occurs anytime new occurrence data are acquired or new biological information about the pest or pathogen is discovered; new data require that model parameters be re‐calibrated, validated, and updated in the database. (2) The Scenario Modeling Loop involves stakeholders defining a management scenario that they want to test together, using a fully calibrated and validated version of the forecast model, to experiment with strategies that they can then implement on the ground as part of real‐world adaptive management; the process repeats as stakeholders compare strategies possible under a realistic financial budget and decide on the optimal outcome. (3) The Field Observation and Scientific Feedback Loop is engaged when stakeholders use the forecast to determine management and monitoring priorities; these new surveys and management actions are recorded in the database, triggering another iteration of the Calibration Loop. The Field Observation and Scientific Feedback Loop can also be triggered when new scientific studies reveal information about species characteristics (eg environmental tolerances or host preferences); this new information is then added to the database, potentially leading to new insights that change model assumptions. Such a change triggers another iteration of the Calibration Loop, and the results of forecasts (and hindcasts) with and without the new knowledge are compared. (4) The Participatory Feedback Loop consists of iterative back‐and‐forth discussion with stakeholders to ensure that research progress matches their needs and vision; stakeholders test and provide input not only on the forecast model but also on ways in which they prefer to interact with it.

To date, we and our collaborators have been using PoPS and the iterative modeling cycle (Figure 3) to forecast the spread of eight emerging pests and pathogens, including *Puccinia striiformis* (wheat stripe rust), *Phytophthora infestans* (late blight), *Lobesia botrana* (European grapevine moth), *Aphthae epizooticae* (foot‐and‐mouth disease), *Alphacoronavirus* (porcine epidemic diarrhea virus), and *Betaarterivirus suid* 1 (porcine respiratory syndrome virus). Below, we describe the use of PoPS in two case studies, spotted lanternfly (SLF) and sudden oak death (SOD), focusing on the Calibration Loop and the value of new data supplied by the Field Observation and Scientific Feedback Loop.

## Case studies: SLF and SOD

SLF and the pathogenic water mold that causes SOD are rapidly emerging threats to natural, economic, and cultural resources in the US, and have motivated the establishment of quarantine zones in highly affected areas. SLF, a nonnative invasive planthopper, has posed a threat to fruit crops in Pennsylvania and neighboring states since 2014 (Urban 2019). SOD is responsible for the deaths of millions of trees in California forests over the past several decades, and the recent introduction of a novel and more aggressive strain in Oregon has prompted renewed concern about economic losses (Gaydos *et al*. 2019). We have been working closely with USDA APHIS (on SLF) and the Oregon Department of Forestry and SOD Mortality Task Force (on SOD) to forecast the spread of these invasive species and to test intervention strategies using PoPS (WebFigures 1 and 2).

In all applications of PoPS, the model is calibrated by comparing observed data to simulated data and setting thresholds so that the model iteratively approaches a best fit. Specifically, we use approximate Bayesian computation with a sequential Markov chain and a multivariate normal perturbation kernel (Filippi *et al*. 2013; Minter and Retkute 2019), an approach that allows calibration of simulation models when a likelihood function is not available. We use Bayesian updating to iteratively update model parameters (β, λ, α_1_, α_2_; see Panel 1 for an explanation of these parameters; Figure 3), and the weights for the prior and calibrated parameters are based on the number of observations that contribute to each dataset. For example, if the model were being calibrated for the year 2017, with 2000 observations, and 2015 and 2016 each had 1000 observations, the prior parameters (from 2015 + 2016 data) and calibrated parameters (for 2017) would each be weighted by 0.5 toward the posterior distribution of our parameter set.

**Figure 4:**
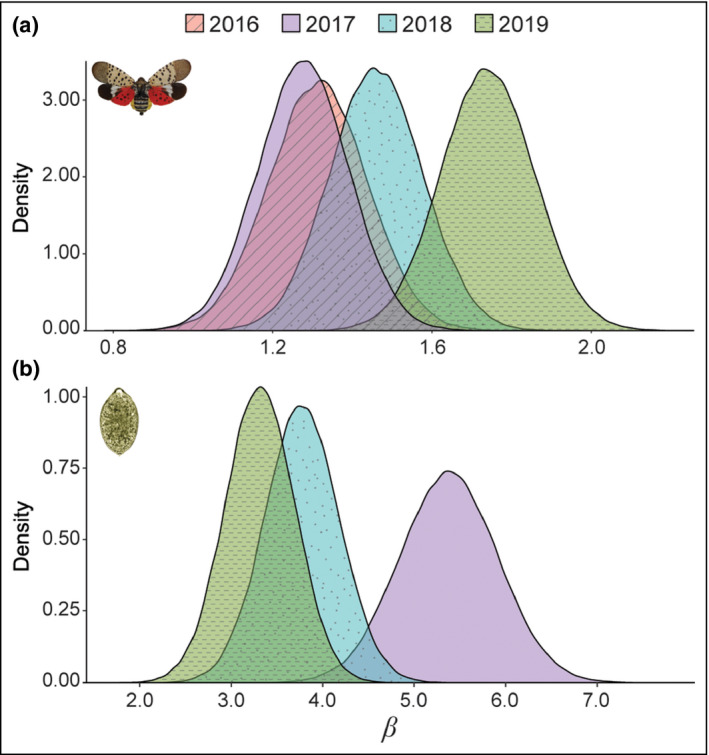
Change in beta (β, pest or pathogen reproductive rate) by species, by calibration year. (a) For SLF, β generally increased over time, most likely due to more intense data collection beginning in 2018. (b) SOD reproductive rate is more variable than SLF; iterative updating with new data therefore permits capturing the breadth of yearly possibilities, moving the parameter set closer toward the mean, and decreasing the potential for outlier years to drive estimates of occurrence. Illustration of SLF and SOD by L Barringer (Pennsylvania DoA) and M Garbelotto (UC Berkeley), respectively.

SLF forecasts were calibrated using 2015–2019 data provided by USDA APHIS and the state‐level agricultural agencies of Pennsylvania, New Jersey, Delaware, Maryland, Virginia, and West Virginia (WebFigures 1 and 2). The distribution of β (pest or pathogen reproduction rate) increased over time (Figure 4a), presumably due to greater, and more targeted, survey effort in later years by field personnel that produced more reliable and comprehensive occurrence data. SOD forecasts were calibrated using 2016–2019 data provided by the Oregon Department of Forestry (WebFigures 1 and 2). Over this time period, the distribution of β decreased and became less uncertain, suggesting that spread in the first year of data was atypical (Figure 4b).

In all applications of PoPS, the model’s predictive skill is validated by hindcasting (ie “forecasting” the past); we ran 100,000 simulations of the model (because it stochastically draws from dispersal distributions) and compared model results with actual observations over the predicted time interval. For example, if we start with 2016 data and calibrate a model to forecast 2017 spread, we then use data collected in 2017 to both (a) forecast 2018 spread and (b) hindcast 2016 and 2017 spread, to see whether recalibration improved model skill. We repeated this process with each set of newly collected data.

The large number of simulations run per time interval enabled us to calculate accuracy assessments. Quantity disagreement, an often‐used assessment, reflects how many cells are predicted differently than observed. However, simulations equally accurate in their predicted *number* of changed cells may predict very different *spatial patterns* of changed cells (eg patchy versus dispersed; see Pickard *et al*. 2017). We therefore also calculated configuration disagreement (Pickard *et al*. 2017) to quantify mismatch between the predicted and observed spatial patterns of infestation. For our stakeholders in invasion management, the ability to understand the *pattern* of spread is more important than the exact location of a new infection. We therefore urge forecasters to adopt validation metrics like configuration disagreement that assess spatial accuracy of forecasts, to provide better utility for on‐the‐ground managers who must decide where to allocate resources.

**Figure 5:**
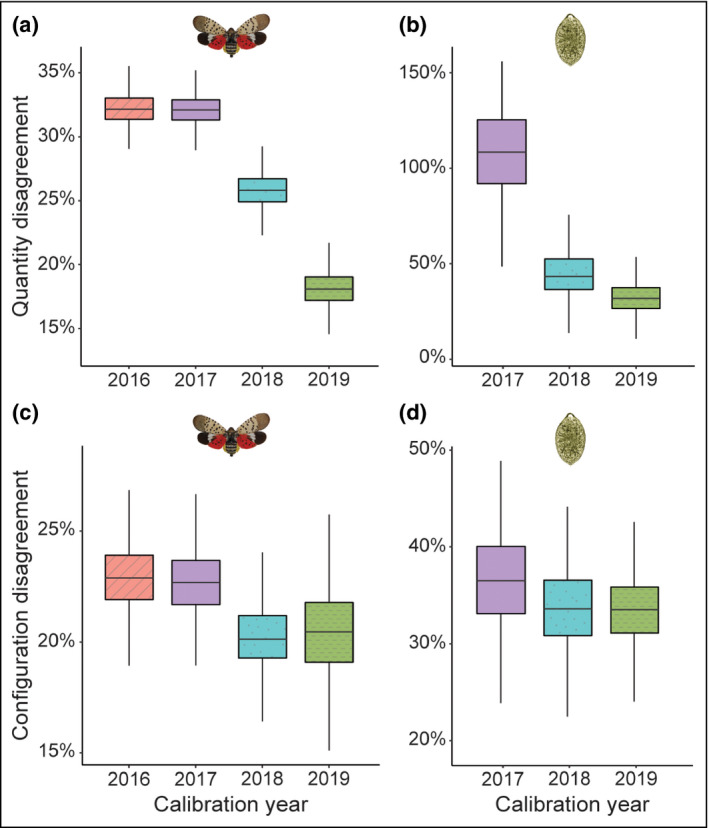
Quantity and configuration disagreement summed for all hindcast years (2016–2019 SLF, 2017–2019 SOD), by species, by calibration year. Quantity disagreement was calculated as the absolute value of predicted occurrences minus observed occurrences, divided by observed occurrences; configuration disagreement was calculated after Pickard *et al*. (2017). Values greater than 100% indicate considerable overprediction. (a) Quantity for SLF; (b) quantity for SOD; (c) configuration for SLF; (d) configuration for SOD. Reductions in disagreement over time indicate improved model skill with iterative calibration using new data. Horizontal lines within boxes depict median values, boxes represent the interquartile range (25th–75th percentiles), and whiskers (vertical lines) represent 1.5×interquartile range. Illustration of SLF and SOD by L Barringer (Pennsylvania DoA) and M Garbelotto (UC Berkeley), respectively.

We iteratively hindcasted the SLF and SOD outbreaks to examine the impact of calibration year on model performance. We hindcasted SLF spread from 1 Jan 2016 to 31 Dec 2019 (Figure 5, a and c) and SOD spread from 1 Jan 2017 to 31 Dec 2019 (Figure 5, b and d), quantifying how well each parameter set matched the observed outbreaks. In both cases, iteratively calibrating the model with more recent data increased hindcast accuracy (that is, reduced disagreement; Figure 5). Many models of pest or pathogen spread are calibrated at a single point in time or for a single epidemic and used to make predictions thereafter without iterative calibration when new data arrives (eg Meentemeyer *et al*. 2011; Fitzpatrick *et al*. 2012; Cunniffe *et al*. 2016). Our results underscore the importance of repeatedly calibrating forecast models with new information to increase their predictive ability.

## Co‐designing the PoPS forecasting system to make meaningful decisions

Notably, both the creation and design of PoPS were motivated and shaped by stakeholders in US state and federal agencies tasked with containing invasive pests and pathogens. Multiple workshops (Participatory Feedback Loops; Figure 3) with personnel involved in field operations (survey and management teams), policy (resource deployment decision makers), and science and technology (economists and data analysts) led to changes in model outputs, as well as in the design and capabilities of the interface. Details regarding those participatory processes and relevant computational science innovations are forthcoming (Gaydos *et al*. in press; Petrasova *et al*. 2020). Briefly, the PoPS framework incorporates decision analytics specifically meant for testing realistic management scenarios (WebFigure 3) and forecasting how spread may change.

On the basis of the guidance from our stakeholders, we enabled PoPS to simulate adaptive management action every year of a forecast (as opposed to only in Year 1; Figure 1), and incorporated different intervention strategies (ie host removal or pesticide/herbicide applications; WebFigure 3) that match real‐world approaches (eg slowing spread, eradicating an invader, or maintaining containment) with simulated monetary costs. Users interact with either a web‐based or tangible surface interface (eg Tonini *et al*. 2017; Petrasova *et al*. 2018; Gaydos *et al*. 2019) to run PoPS, drawing a polygon or placing a physical marker to indicate treatment at a particular place and time. PoPS then predicts how the treatment affects the timing and spatial configuration of spread, comparing the outcome to a scenario with no management.

Once a pest or pathogen has been added to the PoPS database, anyone who accesses the system through its web‐based interface can run a treatment scenario for that species. The user‐friendly interface allows stakeholders to calibrate and validate models, test strategies, and make decisions without needing to interact with the code. This approach has been very well received by management professionals who want to use calibrated, validated science‐based models to inform their efforts but who are not themselves modeling experts (Gaydos *et al*. in press).

Our stakeholders have also expressed appreciation for the spatial assessments of accuracy built into the PoPS framework. Given that *where* to allocate resources is just as important as *when*, reporting forecast configuration accuracy is crucial for making spatially explicit decisions. We suggest that the configuration disagreement metric (described by Pickard *et al*. [2017] to evaluate land‐use/land‐change models) allows stakeholders to better understand how well a forecast predicts spatial patterns as compared to other metrics (eg odds ratio) that indicate simply how many grid cells were predicted correctly versus incorrectly.

Our case studies demonstrate that repeated calibration over time with continuously updated data yields higher forecast skill than using only the earliest‐available data to predict future spread. This result strongly supports the recent appeal for *near‐term*, *iterative* forecasting (Dietze *et al*. 2018), wherein predictions are quickly and repeatedly challenged with new data to improve forecast performance. Iterative calibration and prediction can also help decision makers choose where to collect new data, by refining knowledge of where pests are likely to occur. We found that iterative, near‐term forecasting with PoPS allows our team to optimize system performance and helps our collaborators better target sampling and management for emerging pests and pathogens.

## Conclusion

The PoPS framework is a prime example of an ecological forecasting system that has been both scientifically improved and optimized for real‐world use through sustained participation of management stakeholders in both initial and ongoing development. We urge continued investment in ongoing data collection so that forecasts can be further validated and improved in the future. Such an approach represents a response to the request of Dietze *et al*. (2018) and others (eg the Ecological Forecasting Initiative, https://ecoforecast.org) that ecological forecasts be continuously challenged with new information, thereby improving predictions and decision making over management‐relevant timescales.

## Supporting information

Fig S1

Fig S2

Fig S3
