# Supplementary material for: Iteratively forecasting biological invasions with PoPS and a little help from our friends
Source: Front Ecol Environ. 2021 Jun 3;19(7):411–8. doi: 10.1002/fee.2357 (PMC8453564; doi:10.1002/fee.2357)
Supplement: Supplementary file 1 — Fig S1 [file FEE-19-411-s002.pdf]

C Jones *et al*. – Supporting Information

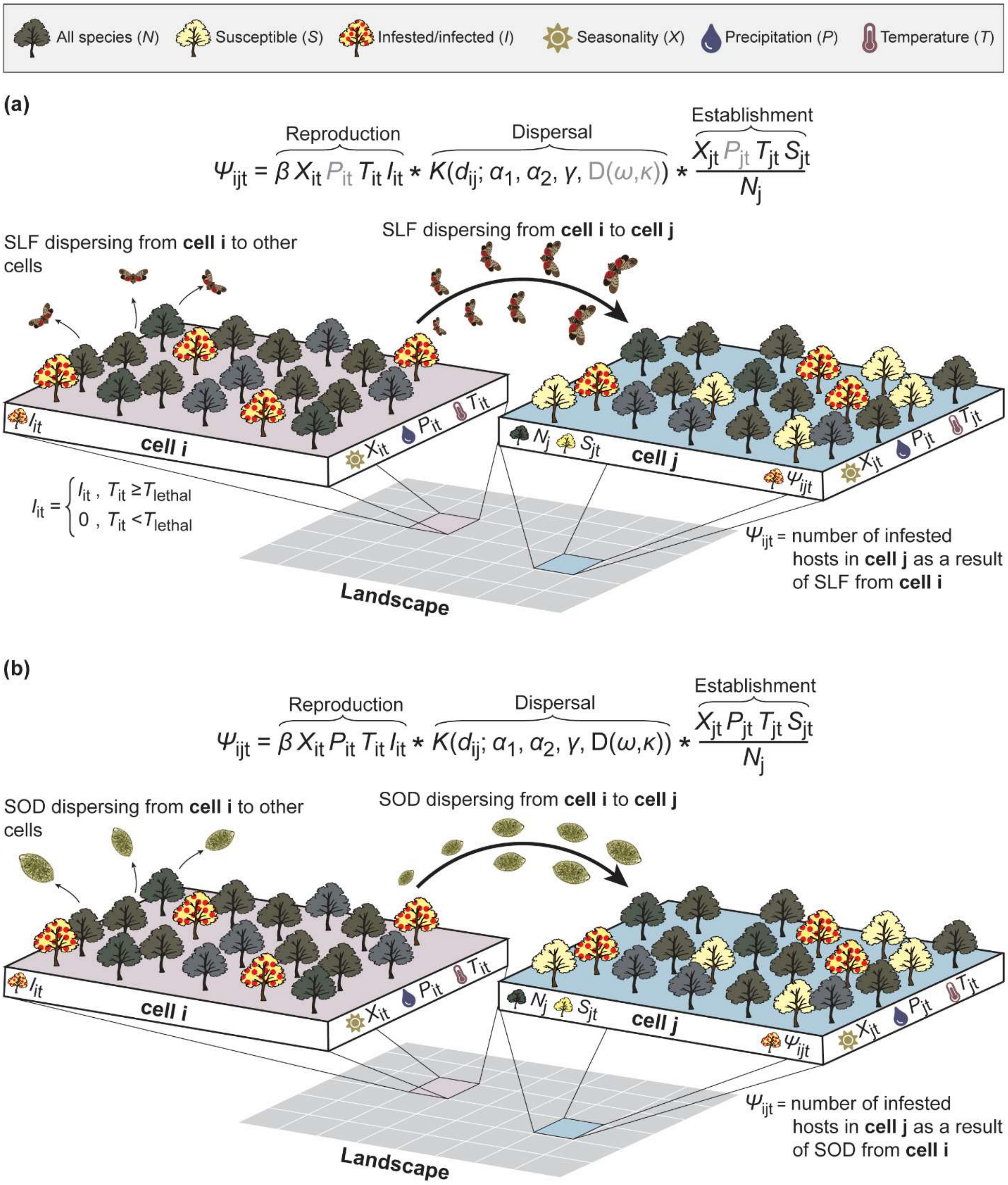

WebFigure 1. Specific model structure for (a) spotted lanternfly (SLF, *Lycorma delicatula*) and (b) the water mold that causes sudden oak death (SOD, *Phytophthora ramorum*). (a) For SLF, we forecast spread across Pennsylvania; New Jersey; Delaware; Maryland; West Virginia; Virginia;

and Washington, DC, at 1-km resolution. Precipitation is not known to have an effect on SLF reproduction or survival, but cold temperatures suppress reproduction, and if the mean temperature in January drops below 12.87°C in a cell, all infestation is removed. SLF spread occurs from May to November. We used MaxEnt to create a tree of heaven (*Ailanthus altissima*) distribution map based on field observations from our stakeholders. (b) For SOD, we forecast spread in one county in Oregon at 100-m resolution. Spread can occur year-round, and is influenced by both temperature and precipitation, along with predominant wind direction and strength. We used Landscape Ecology, Modeling, Mapping, and Analysis (LEMMA) data (Ohmann *et al.* 2011) on tanoak (*Notholithocarpus densiflorus*) distribution, scaled from 30-m to 100-m resolution, as our host species inputs to the model.

WebReference

Ohmann JL, Gregory MJ, Henderson EB, and Roberts HM. 2011. Mapping gradients of community composition with nearest-neighbour imputation: extending plot data for landscape analysis. *J Veg Sci* **22**: 660–76.
