# Supplementary material for: Iteratively forecasting biological invasions with PoPS and a little help from our friends
Source: Front Ecol Environ. 2021 Jun 3;19(7):411–8. doi: 10.1002/fee.2357 (PMC8453564; doi:10.1002/fee.2357)
Supplement: Supplementary file 2 — Fig S2 [file FEE-19-411-s003.pdf]

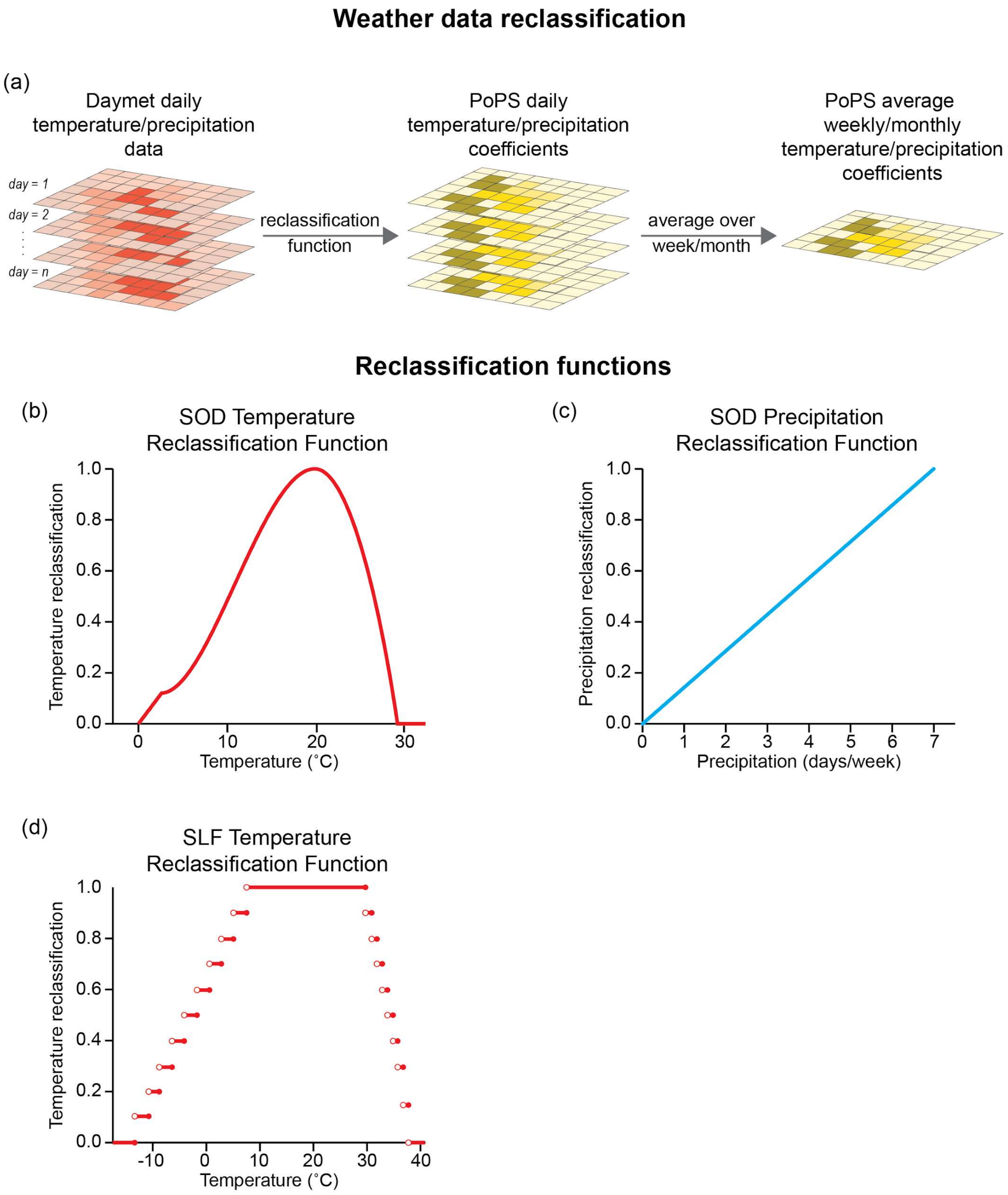

WebFigure 2. Reclassification of weather data to species-specific survival and reproduction coefficients. (a) To run the spread model in PoPS, all raw temperature and precipitation values

were first converted to indices ranging from 0–1 to describe their impact on a species' ability to survive and reproduce; the transformations, tailored for each species, were saved in the PoPS database. For SOD, daily Daymet (Thornton *et al.* 2017) (b) temperature and (c) precipitation were converted to weekly weather coefficients ranging from 0–1 (Meentemeyer *et al.* 2011). For SLF, daily Daymet temperature (d) was converted into a monthly coefficient ranging from 0–1 (based on conversations with field operations personnel).

WebReferences

Meentemeyer RK, Cunniffe NJ, Cook AR, *et al*. 2011. Epidemiological modeling of invasion in heterogeneous landscapes: spread of sudden oak death in California (1990–2030). *Ecosphere* **2**: 17.

Thornton PE, Thornton MM, Mayer BW, *et al.* 2017. Daymet: daily surface weather data on a 1-km grid for North America, v3. Oak Ridge, TN: Oak Ridge National Laboratory Distributed Active Archive Center.
