# Supplementary material for: Iteratively forecasting biological invasions with PoPS and a little help from our friends
Source: Front Ecol Environ. 2021 Jun 3;19(7):411–8. doi: 10.1002/fee.2357 (PMC8453564; doi:10.1002/fee.2357)
Supplement: Supplementary file 3 — Fig S3 [file FEE-19-411-s001.pdf]

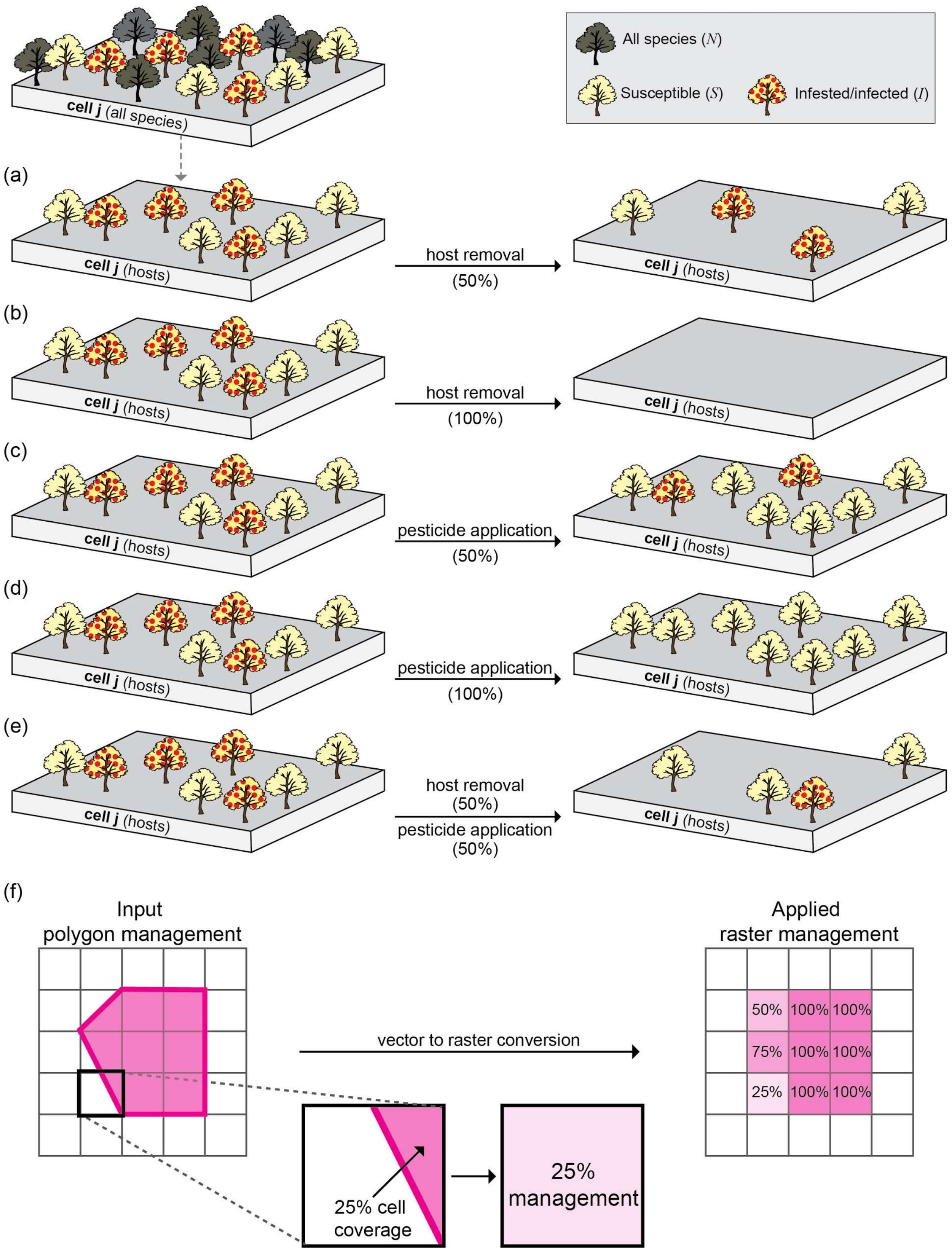

WebFigure 3. Description of PoPS management types. PoPS allows two types of management: (a and b) host removal and (c and d) pesticide application. (e) The model also permits both

management types to be applied simultaneously. (f) Management input as polygons is converted to raster management based on percent cell coverage. The “% removal” applied (a–e) is multiplied by the percent of the cell covered by the management (f) for the overall percentage applied in a cell; for example, using the 25% management cell in (f) and the 50% management in (a) would result in 12.5% of the cell being managed.
